# Energy-efficient control of a screw-drive pipe robot with consideration of actuator’s characteristics

**DOI:** 10.1186/s40638-016-0045-z

**Published:** 2016-07-11

**Authors:** Peng Li, Shugen Ma, Congyi Lyu, Xin Jiang, Yunhui Liu

**Affiliations:** School of Mechanical Engineering and Automation, Harbin Institute of Technology Shenzhen Graduate School, ShenZhen, 518055 China; Department of Mechanical and Automation Engineering, The Chinese University of Hong Kong, Shatin, Hong Kong China; Shenyang Institute of Automation, Chinese Academy of Sciences, Shenyang, 110016 China; Department of Robotics, Ritsumeikan University, Shiga-ken, 525-8577 Japan

**Keywords:** In-pipe robot, Energy-efficient control, Optimal velocity strategy

## Abstract

Pipe robots can perform inspection tasks to alleviate the damage caused by the pipe problems. Usually, the pipe robots carry batteries or use a power cable draining power from a vehicle that has many equipments for exploration. Nevertheless, the energy is limited for the whole inspection task and cannot keep the inspection time too long. In this paper, we use the total input energy as the cost function and a more accurate DC motor model to generate an optimal energy-efficient velocity control for a screw-drive pipe robot to make use of the limited energy in field environment. We also propose a velocity selection strategy that includes the actual velocity capacity of the motor, according to the velocity ratio $k_{\mathrm{v}}$, to keep the robot working in safe region and decrease the energy dissipation. This selection strategy considers three situations of the velocity ratio $k_{\mathrm{v}}$ and has a wide range of application. Simulations are conducted to compare the proposed method with the sinusoidal control and loss minimization control (minimization of copper losses of the motor), and results are discussed in this paper.

## Background

With the advancement of the robotics and industrial technology, many pipe robots have been developed to explore the pipes that have cracks or defects to avoid serious accidents [1–4].

Up to now, there are more focus on the energy-efficient control of robots [5, 6]. Robots that perform pipe inspection task are often in the field environment, and the energy is a crucial limitation to the time of execution of a task. Most of the pipe robots are driven by DC motors. If the energy-efficient method is applied to the pipe inspection system, the energy dissipation will be decreased and the total time of performing a task will be increased. The energy dissipated through many ways, but only controlling the armature current and field current losses is feasible [7, 8]; further, many researchers conducted on the loss minimization control of the DC motor [9, 10]. They use the armature resistance loss and field resistance loss as the performance index to reduce the energy dissipation, and get the optimal control law, but in the view of the total input energy that is drawn from the power source is usually not optimal.

This paper proposes an energy-efficient solution for the control of a screw-type pipe robot by using an improved DC motor model and employing the total input energy as the performance index that reflect the whole system energy consumption. Straight pipe structure is the most common type; thus, this paper is limited to discuss the condition that the pipe robot is used in the straight horizontal pipe. Additionally, sinusoidal fashion control and the loss minimization control that only considers armature resistance are used as the comparison methods.

## Methods

### The screw-drive pipe robot

**Fig. 1:**
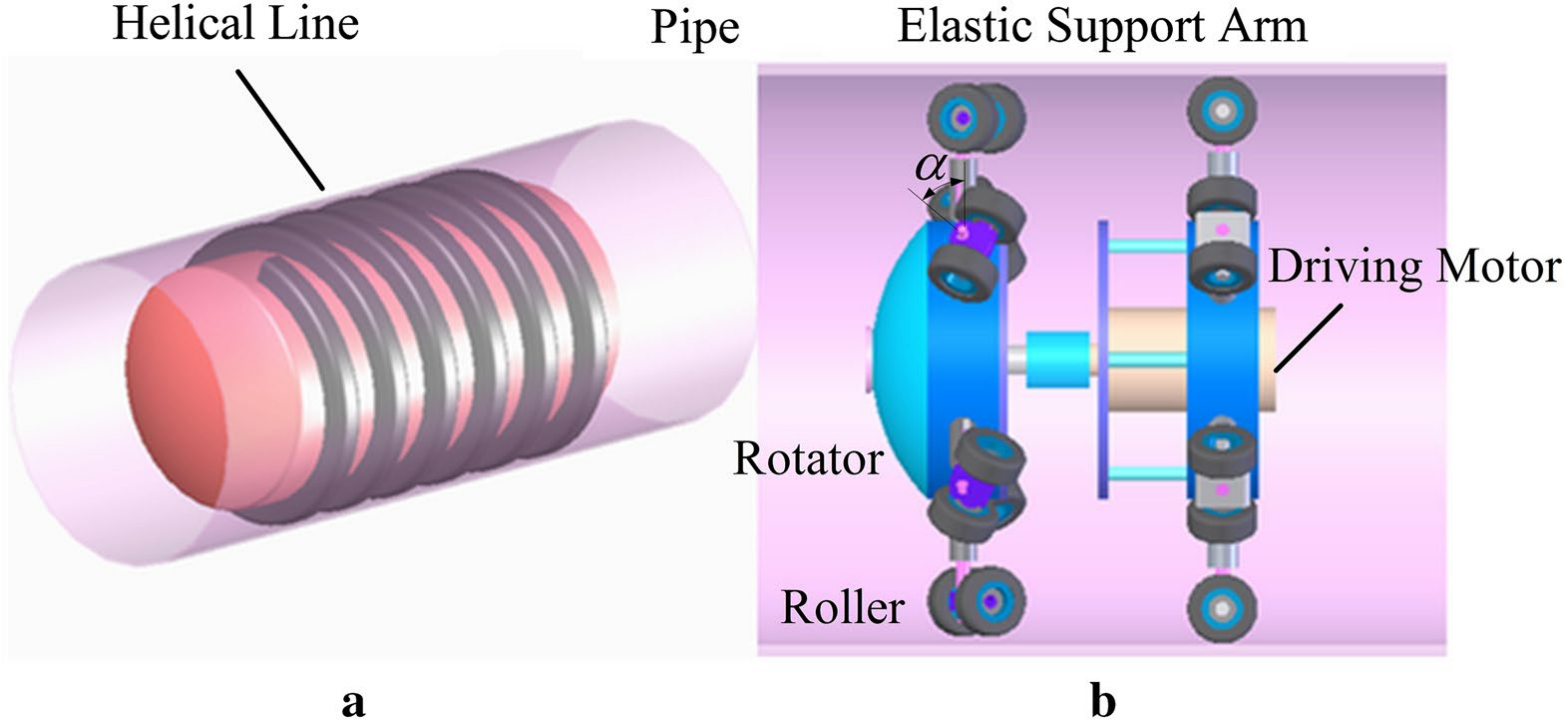
The screw-type pipe robot. **a** Sketch of the driving principle, **b** typical screw-drive-type robot

The environment of the pipe is not invariable; thus, the pipe robot should possess the characteristics of multifunction, adaptability and efficiency. The screw-drive robots are not rare [3, 4], and driving principle is illustrated in Fig. 1.

Typical screw-drive robot is usually composed of a rotator, elastic support arms, rollers and a motor for driving. The rollers have a constant incline angle with respect to the cross section of the pipe. When the motor turns, the whole body moves forward. If the motor turns reversely, the body moves backward. To propel a screw-drive-type robot, one motor is enough for straight pipe and elbow. However, for the T-shape pipe, extra navigation mechanism is needed.

**Fig. 2:**
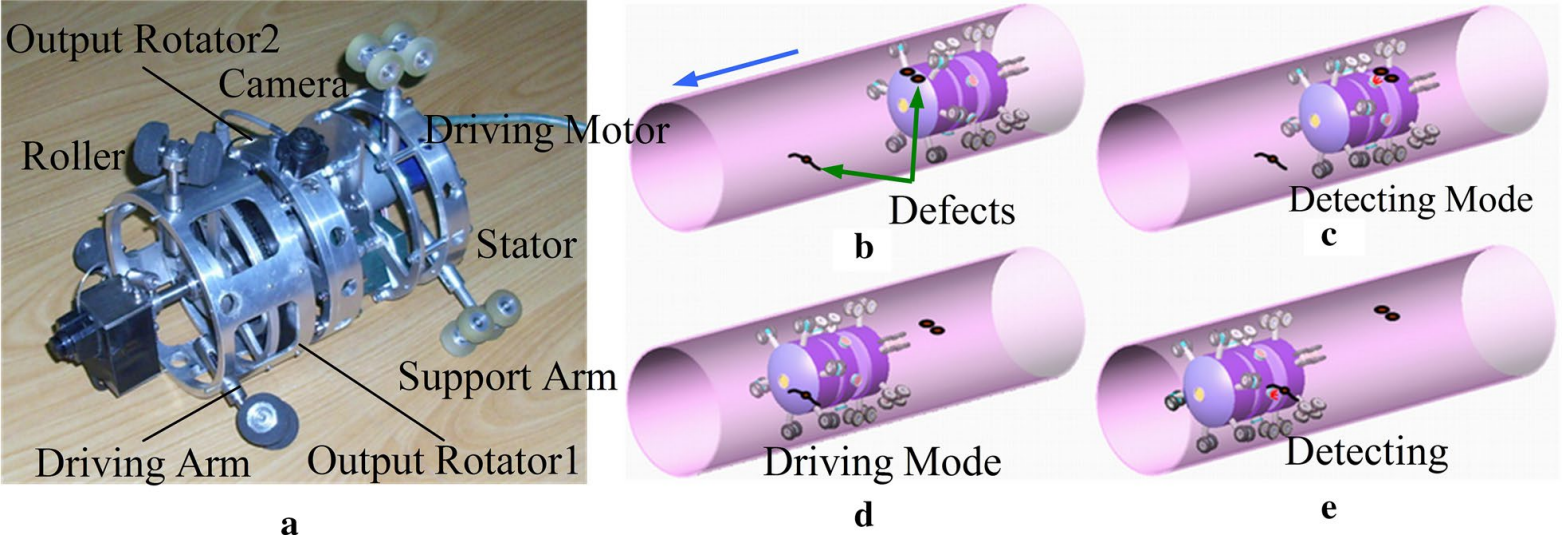
The screw-drive robot with driving and detecting ability with one DC motor. **a** Prototype, **b** robot is under driving mode, **c** the solenoid is electrified, and robot is under detecting mode and check the defect of the pipe, **d** after checking the area, the solenoid is set off, and the robot is back to driving mode again, **e** another operation cycle of detecting

A screw-type robot considered in this paper is shown in Fig. 2a. The advantage of this robot is that it has mobile ability in the pipe and detecting function for inspection, while only one DC motor is installed, which results in low energy consumption and low cost to fabricate. The robot has two working modes: a driving mode and a detecting mode. The robot propels itself in the pipe under the driving mode, and it is used for finding the defect of the pipe under the detecting mode. By setting an on–off solenoid, the two working modes will switch to each other; thus, the robot performs the inspection task without other extra motors. Therefore, such a robot is an efficient design, because it uses one motor and a solenoid to perform a task instead of two motors (one motor for moving and the other motor for detecting). There are two types of driving arm: One type has a constant incline angle as shown in Fig. 1b, while the incline angle of the other type changes according to the payload variation. The two types of driving arm can be both fixed on the robot, but this paper only considers the driving arms with constant incline angle. Further detailed information, for example, the mechanical structure, can be found in [4].

### Motion equation of the DC motor

#### Basic equations of the DC motor

**Fig. 3:**
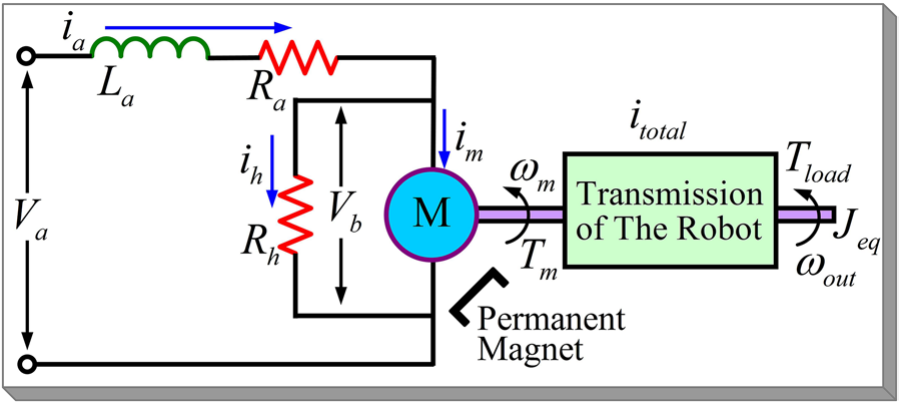
Equivalent circuit of the DC motor

An armature-controlled DC motor is shown in Fig. 3, in which the field current is constant.

The basic equations of circuit are

1 $$\begin{aligned}&{L_{\mathrm{a}}}\frac{{\mathrm{d}{i_{\mathrm{a}}}}}{{\mathrm{d}t}} + {R_{\mathrm{a}}}{i_{\mathrm{a}}} + {V_{\mathrm{b}}} = {V_{\mathrm{a}}} \end{aligned}$$

2 $$\begin{aligned}&{i_{\mathrm{a}}} = {i_{\mathrm{m}}} + {i_{\mathrm{h}}} \end{aligned}$$

${L_{\mathrm{a}}}$ is the armature inductance (the voltage generated by ${L_{\mathrm{a}}}$ is much smaller than that of ${R_{\mathrm{a}}}$ and ${V_{\mathrm{b}}}$; thus, in this paper we do not consider the influence of ${L_{\mathrm{a}}}$, ${V_{\mathrm{a}}}$ and ${V_{\mathrm{b}}}$ is the applied armature voltage and back-emf, respectively. ${R_{\mathrm{a}}}$ is the armature resistance, while ${R_{\mathrm{h}}}$ is equivalent resistance for power losses due to the air resistance of rotor and power loss due to the friction between mechanical parts, and Ma had pointed out that the ${R_{\mathrm{h}}}$ should be included when the motor efficiency is calculated [11]. ${i_{\mathrm{a}}}$, ${i_{\mathrm{h}}}$ and ${i_{\mathrm{m}}}$ are the current of the ${R_{\mathrm{a}}},\,{R_{\mathrm{h}}}$ and the armature, respectively. And the back-emf ${V_{\mathrm{b}}}$ and the armature torque and current are given by

3 $$\begin{aligned} {V_{\mathrm{b}}}= {K_{\mathrm{e}}}{\omega _{\mathrm{m}}} \end{aligned}$$

4 $$\begin{aligned} {T_{\mathrm{m}}}= {K_{\mathrm{t}}}{i_{\mathrm{m}}} \end{aligned}$$

5 $$\begin{aligned} {i_{\mathrm{m}}}= {i_{\mathrm{a}}} - {V_{\mathrm{b}}}{/}{R_{\mathrm{h}}} \end{aligned}$$

where ${K_{\mathrm{e}}}$ and ${K_{\mathrm{t}}}$ is the back-emf constant and torque constant, respectively, and they have the same value, when SI units are used.

By using the above equation, we can also calculate the value of ${R_{\mathrm{h}}}$,

6 $$\begin{aligned} {R_{\mathrm{h}}}=\frac{V_{\mathrm{b}}}{i_{\mathrm{a}}-i_{\mathrm{m}}}=\frac{{K_{\mathrm{e}}}{\omega _{\mathrm{m}}}{R_{\mathrm{a}}}}{{V_{\mathrm{b}}}-{K_{\mathrm{e}}}{\omega _{\mathrm{m}}}-{R_{\mathrm{a}}}{i_{\mathrm{m}}}} \end{aligned}$$

As shown in Fig. 3, the whole robot has been treated as a power transmission with a gear ratio of ${i_\mathrm{total}}$; as a result, it amplify the motor torque ${T_{\mathrm{m}}}$ and angular velocity ${\omega _{\mathrm{m}}}$ into that of the output rotator ${T_{\mathrm{out}}}$ and ${\omega _{{\mathrm{out}}}}$ and conquer the payload ${T_{\mathrm{load}}}$. Thus, the dynamics of the motor is

7 $$\begin{aligned} {J_{\mathrm{eq}}}\frac{{\mathrm{d}{\omega _{\mathrm{m}}}}}{{\mathrm{d}t}} + {c_{\mathrm{m}}}{\omega _{\mathrm{m}}} + \frac{{{T_{\mathrm{load}}}}}{{{i_{\mathrm{total}}}}} = {T_{\mathrm{m}}} \end{aligned}$$

${c_{\mathrm{m}}}$ is the viscous coefficient of the motor, and ${T_{\mathrm{load}}}$, ${J_{\mathrm{eq}}}$ are the load torque and equivalent moment of inertia of the robot’s output rotator and the rotor of the motor, respectively.

#### Efficiency of the DC motor

From the above equations, the mechanical power generated by the motor is ${T_{\mathrm{m}}}{\omega _{\mathrm{m}}}$, and the motor efficiency ${\eta _{\mathrm{motor}}}$ is

8 $$\begin{aligned} {\eta _{\mathrm{motor}}} =\frac{{T_{\mathrm{m}}}{\omega _{\mathrm{m}}}}{{V_{\mathrm{a}}}{i_{\mathrm{a}}}} \end{aligned}$$

**Fig. 4:**
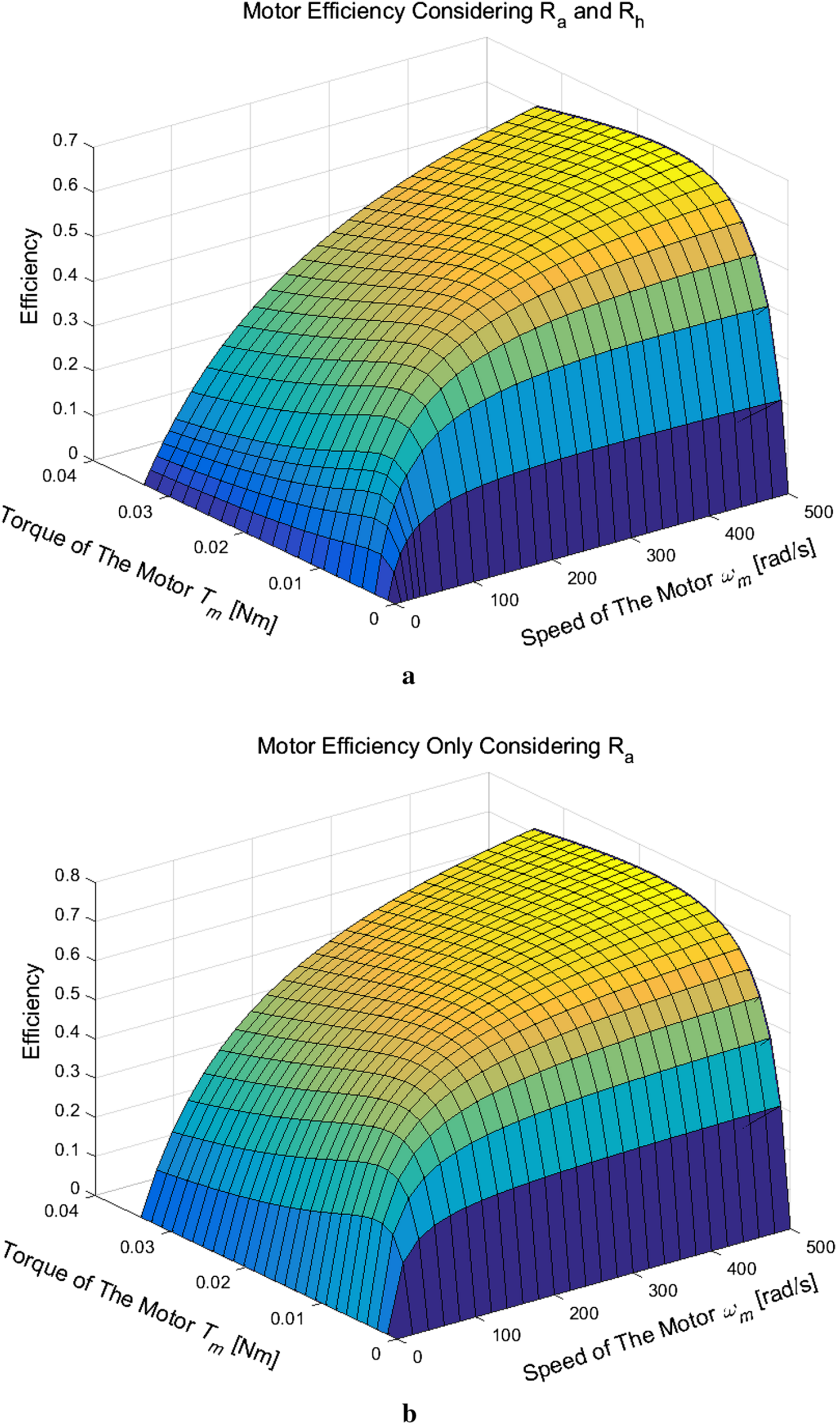
Efficiency of an armature-controlled DC motor. **a** Motor efficiency that considering ${R_{\mathrm{a}}}$ and ${R_{\mathrm{h}}}$, **b** motor efficiency that only considering ${R_{\mathrm{a}}}$

The above equations have shown the influence of ${R_{\mathrm{h}}}$, when calculating the motor efficiency. Moreover, using the above equations, the motor efficiency that is considering ${R_{\mathrm{a}}}$ and ${R_{\mathrm{h}}}$ and that of only considering ${R_{\mathrm{a}}}$ is shown in Fig. 4 with the same parameters in Table 1. The reason of Fig. 4’s result is that considering ${R_{\mathrm{a}}}$ and ${R_{\mathrm{h}}}$ needs more electrical energy than that of only considering ${R_{\mathrm{a}}}$, when the motor working at a same combination of torque and velocity. The motor efficiency has decreased, when ${R_{\mathrm{h}}}$ is considered.

#### Motion and force analysis of the robot

The kinematics of the wheel-type robot is generally calculated by

9 $$\begin{aligned} v = {\omega _{\mathrm{out}}}{\gamma } \end{aligned}$$

while *v* and ${\omega _{\mathrm{out}}}$ are the translational speed and rotational speed of robot, respectively. ${\gamma }$, here, is a coefficient that converts rotational speed into translational speed. Moreover, the kinematics of screw-drive robot is (detailed can be found in [3, 4])

10 $$\begin{aligned} v= \, & {} {\omega _{\mathrm{out}}}\left( {{r_\mathrm{w}} + L} \right) \tan \alpha \nonumber \\ \gamma= \,& {} \left( {{r_\mathrm{w}} + L} \right) \tan \alpha \end{aligned}$$

**Fig. 5:**
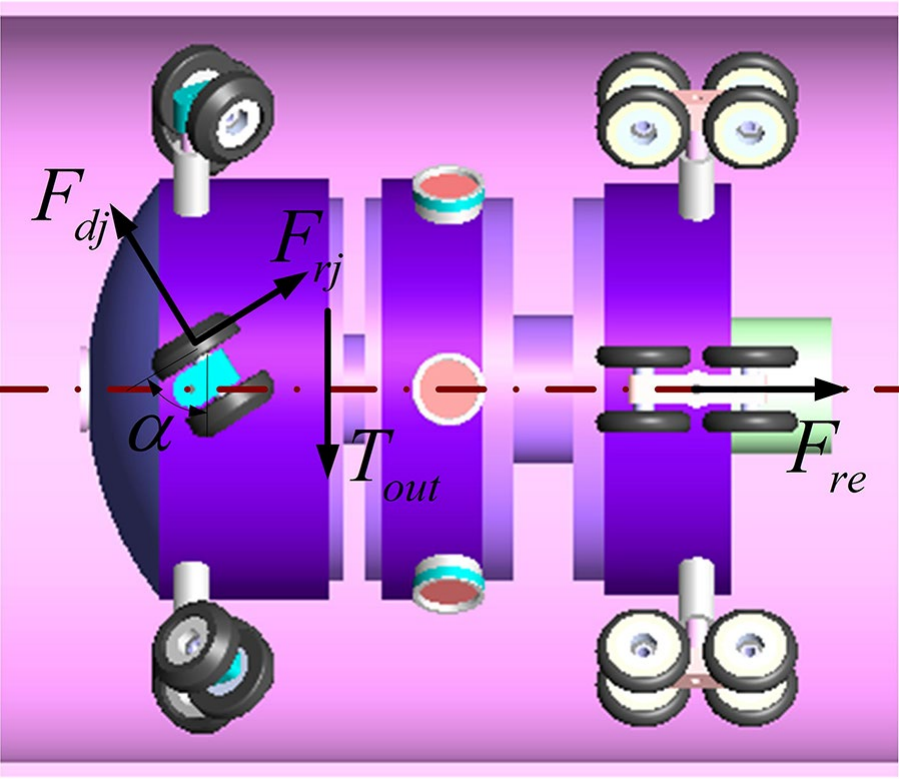
Forces acting on the robot

${r_\mathrm{w}}$ is the radius of the roller and $L = 0.5D - {r_\mathrm{w}}$, while *D* is the inner diameter of the pipe; $\alpha$ is the constant incline angle, as shown in Fig. 5.

Let ${F_{dj}}$ and ${F_{rj}}$ denote the normal and tangential friction, and ${f_\mathrm{n}}$ and ${f_{\mathrm{t}}}$ be the corresponding frictional coefficients, respectively. ${F_{\mathrm{re}}}$ is all the other resistant forces acting on the robot, and *n* is the number of the driving arms. The static equilibrium equations can be derived from Fig. 5

11 $$\begin{aligned}&n\left( {{F_{dj}}\cos \alpha - {F_{rj}}\sin \alpha } \right) = {F_{\mathrm{re}}} \end{aligned}$$

12 $$\begin{aligned}&n\left( {{F_{dj}}\sin \alpha + {F_{rj}}\cos \alpha } \right) = 2{T_{\mathrm{out}}}/D \end{aligned}$$

From (11)and (12), the relation between ${T_{\mathrm{load}}}$ and ${T_{\mathrm{out}}}$ is

13 $$\begin{aligned} {T_{\mathrm{load}}} = \frac{{{F_{dj}}\cos \alpha - {F_{rj}}\sin \alpha }}{{{F_{dj}}\sin \alpha + {F_{rj}}\cos \alpha }}{T_{\mathrm{out}}} =F\left( \alpha \right) {T_{\mathrm{out}}} \end{aligned}$$

**Fig. 6:**
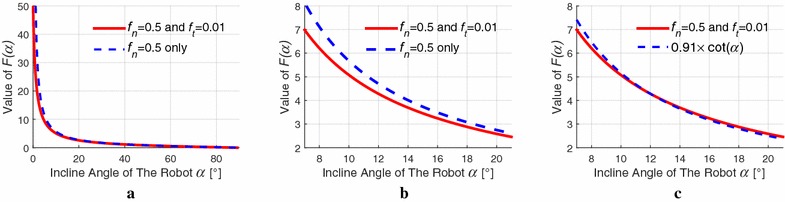
Values of $F(\alpha )$. **a** ${f_n}=0.5$ and ${f_t}=0.01$ versus ${f_n}=0.5$ only. **b** Zoom in figure of (**a**) with the interval from ${8^\circ}$ to ${20^\circ}$. **c** ${f_n}=0.5$ and ${f_t}=0.01$ versus ${f_n}=0.5$ and an artificial coefficient 0.91 times (13)

Thus, the load torque term ${T_{\mathrm{load}}}/{i_{\mathrm{total}}}$ can be updated by ${{{T_{\mathrm{load}}}}}/{{F\left( \alpha \right) {i_{\mathrm{total}}}}}$. Now, we consider the condition of not including the tangential friction force. Figure 6a, b shows the value of $F(\alpha )$ with environment parameters ${f_\mathrm{n}} =0.5$ and ${f_{\mathrm{t}}}=0.01$ and that of ${f_\mathrm{n}}=0.5$ and ${f_{\mathrm{t}}} =0$. As shown in Fig. 6c, the difference of the two conditions is almost the same after an artificial coefficient 0.91 multiplies ${F(\alpha )}$ between 9° and 20°, under the condition ${f_\mathrm{n}}=0.5$ and ${f_{\mathrm{t}}}=0.$

Now, $F(\alpha )$ can be simplified as follows

14 $$\begin{aligned} F\left( \alpha \right) = 0.91\cot \alpha \end{aligned}$$

while the 0.91 is the artificial coefficient to calculate $F(\alpha )$ between the angle of 9° and 20°. The motion equation is derived

15 $$\begin{aligned} {J_{\mathrm{eq}}}{\dot{\omega } _{\mathrm{m}}} + \left( {\frac{{{K_{\mathrm{t}}}{K_{\mathrm{e}}}}}{{{R_{\mathrm{h}}}}} + {c_{\mathrm{m}}}} \right) {\omega _{\mathrm{m}}} + \frac{{{T_{\mathrm{load}}}}}{{{i_{\mathrm{total}}}F\left( \alpha \right) }} - {K_{\mathrm{t}}}{i_{\mathrm{a}}} = 0 \end{aligned}$$

and the state equation is given by

16 $$\begin{aligned} {\dot{\omega } _{\mathrm{m}}} = - A{\omega _{\mathrm{m}}} + B{i_{\mathrm{a}}} + C \end{aligned}$$

where $${A = \frac{{{K_{\mathrm{t}}}{K_{\mathrm{e}}} + {c_{\mathrm{m}}}{R_{\mathrm{h}}}}}{{{J_{\mathrm{eq}}}{R_{\mathrm{h}}}}}}$$, $${B = \frac{{{K_{\mathrm{t}}}}}{{{J_{\mathrm{eq}}}}}}$$, $${C = - \frac{{{T_{\mathrm{load}}}}}{{F\left( \alpha \right) {J_{\mathrm{eq}}}{i_{\mathrm{total}}}}}}$$.

### Energy-efficient control

According to the task requirements, this robot has two modes:

1. Cruise start/stop mode: Cruise start mode is used to start the motor and the robot at a specified speed; then, the motor and the robot keep this speed moving to find the potential defect of the pipe. When the detecting camera finds the suspicious defect, the robot will stop and detect that area carefully; thus, stopping the robot is called cruise stop mode.
2. Location mode: Sometimes, a segment of pipe need not to be checked; thus, the robot just passes by. The operator only inputs the displacement $S_{\mathrm{f}}$ within the time $t_{\mathrm{f}}$; then, the optimal velocity profile is generated according to the value of $k_{\mathrm{v}}$, which is a speed ratio defined in (38).

#### Cruise start/stop mode

Under this mode, the final velocity ${\omega _{\mathrm{f}}}$ and the corresponding time ${t_{\mathrm{f}}}$ are given, and the optimal velocity profile is generated by the control law. After the time ${t_{\mathrm{f}}}$, the actuator of the robot will keep the value of ${\omega _{\mathrm{f}}}$ moving forward, since keeping the velocity invariant will result in the minimization of the extra consumption of the total input electrical energy. On the contrary, under the cruise stop mode, the initial motor velocity ${\omega _0}$ and the time ${t_{\mathrm{f}}}$ are given.

The above problem is to find the optimal control ${i_{\mathrm{a}}}$ that minimizes the cost function for the given constrains. The cost function is the integrals of the total electrical energy

17 $$\begin{aligned} {\mathrm{min }}\,{E_{\mathrm{in}}} = \int _0^{{t_{\mathrm{f}}}} {{V_{1,\mathrm{a}}}{i_{1,\mathrm{a}}}\mathrm{d}t} \end{aligned}$$

with the constrains

$$\begin{aligned} {{\dot{\omega }_{1,\mathrm{m}}} = - A{\omega _{1,\mathrm{m}}} + B{i_{1,\mathrm{a}}} + C} \end{aligned}$$

The Hamiltonian is formed by

18 $$\begin{aligned} H = ({R_{\mathrm{a}}}{i_{1,\mathrm{a}}} + {K_{\mathrm{e}}}{\omega _{1,\mathrm{m}}}){i_{1,\mathrm{a}}} + {\lambda _1}\left( { - A{\omega _{1,\mathrm{m}}} + B{i_{1,\mathrm{a}}}} \right) \end{aligned}$$

with the costate equation as

19 $$\begin{aligned} {\dot{\lambda }_1} = - \frac{{ \partial H}}{{ \partial {\omega _{1,\mathrm{m}}}}} \end{aligned}$$

where the subscript 1 represents the corresponding variations of the cruise start/stop mode. The optimal control is obtained by

20 $$\begin{aligned} {\left. {\frac{{ \partial H}}{{ \partial {i_{1,\mathrm{a}}}}}} \right| _{{i_{1,\mathrm{a}}} = i_{1,\mathrm{a}}^ * }} = 0 \end{aligned}$$

and solving for $i_{1,\mathrm{a}}^ *$

21 $$\begin{aligned} i_{1,\mathrm{a}}^ * = \frac{{ - 1}}{{2{R_{\mathrm{a}}}}}\left( {{\lambda _1}B + {K_{\mathrm{e}}}{\omega _{1,\mathrm{m}}}} \right) \end{aligned}$$

Solve the differential equations, and the optimal velocity is formulated by

22 $$\begin{aligned} {\omega _{1,\mathrm{m}}}\left( t \right) = {C_{11}}{e^{t/{\tau _{\mathrm{m}}}}} + {C_{12}}{e^{ - t/{\tau _{\mathrm{m}}}}} + {F_{0,1}} \end{aligned}$$

${\tau _{\mathrm{m}}} = {\sqrt{{A^2} + AB{K_{\mathrm{e}}}/{R_{\mathrm{a}}}} ^{ - 1}},{F_{0,1}} = - \left( {2AC{R_{\mathrm{a}}} + CB{K_{\mathrm{e}}}} \right) /2{R_{\mathrm{a}}}$ where ${C_{11}}$ and ${C_{12}}$ are constants that are determined by the boundary conditions, and ${\tau _{\mathrm{m}}}$ represents the mechanical time constant. The boundary condition of cruise start mode is $$\begin{aligned}{\omega _{1,\mathrm{m}}}(0)=0, {\omega _{1,\mathrm{m}}}\left( {{t_{\mathrm{f}}}} \right) = {\omega _{\mathrm{f}}}\end{aligned}$$. Then ${C_{11}}$ and ${C_{12}}$ in this condition are

23 $$\begin{aligned} \begin{array}{l} {C_{11}}=\frac{{{\omega _{\mathrm{f}}} + {F_{0,1}}\left( {{e^{ - {t_{\mathrm{f}}}/{\tau _{\mathrm{m}}}}} - 1} \right) }}{{{e^{{t_{\mathrm{f}}}/{\tau _{\mathrm{m}}}}} - {e^{ - {t_{\mathrm{f}}}/{\tau _{\mathrm{m}}}}}}}\\ {C_{12}}=\frac{{ - {\omega _{\mathrm{f}}} - {F_{0,1}}\left( {{e^{{t_{\mathrm{f}}}/{\tau _{\mathrm{m}}}}} - 1} \right) }}{{{e^{{t_{\mathrm{f}}}/{\tau _{\mathrm{m}}}}} - {e^{ - {t_{\mathrm{f}}}/{\tau _{\mathrm{m}}}}}}} \end{array} \end{aligned}$$

While the boundary condition of the cruise stop mode is ${\omega _{1,\mathrm{m}}}\left( 0 \right) = {\omega _0}, {\omega _{1,\mathrm{m}}}\left( {{t_{\mathrm{f}}}} \right) = 0$, then, ${C_{11}}$ and ${C_{12}}$ are

24 $$\begin{aligned} {C_{11}} &= \frac{{ - {\omega _0}{e^{ - {t_{\mathrm{f}}}/{\tau _{\mathrm{m}}}}} - {F_{0,1}}\left( {1 - {e^{ - {t_{\mathrm{f}}}/{\tau _{\mathrm{m}}}}}} \right) }}{{{e^{{t_{\mathrm{f}}}/{\tau _{\mathrm{m}}}}} - {e^{ - {t_{\mathrm{f}}}/{\tau _{\mathrm{m}}}}}}}\nonumber \\ {C_{12}} &= \frac{{{\omega _0}{e^{{t_{\mathrm{f}}}/{\tau _{\mathrm{m}}}}} - {F_{0,1}}\left( {{e^{{t_{\mathrm{f}}}/{\tau _{\mathrm{m}}}}} - 1} \right) }}{{{e^{{t_{\mathrm{f}}}/{\tau _{\mathrm{m}}}}} - {e^{ - {t_{\mathrm{f}}}/{\tau _{\mathrm{m}}}}}}} \end{aligned}$$

The optimal velocity profile of the cruise start/stop mode is derived. Moreover, the optimal control $i_{1,\mathrm{a}}^ *$ is given by

25 $$\begin{aligned} i_{1,\mathrm{a}}^ * = {B^{ - 1}}\left( {{{\dot{\omega } }_{\mathrm{m}}} + A{\omega _{\mathrm{m}}} - C} \right) \end{aligned}$$

#### Location mode

Since many segments of the pipe have been checked and do not need to be checked again, the robot needs position to position control. Under the location mode, the operator inputs the desired displacement ${S_{\mathrm{f}}}$ that the robot will move and the time ${t_{\mathrm{f}}}$ that robot will cost. Similarly, the cost function is also the instantaneous total electrical power

26 $$\begin{aligned} {\mathrm{min }}\,\,{E_{\mathrm{in}}} = \int _0^{{t_{\mathrm{f}}}} {{V_{2,\mathrm{a}}}{i_{2,\mathrm{a}}}\mathrm{d}t} \end{aligned}$$

with the constrains

27 $$\begin{aligned} {\dot{\theta }_{2,\mathrm{m}}}= \, & {} {\omega _{2,\mathrm{m}}}\end{aligned}$$

28 $$\begin{aligned} {\dot{\omega } _{2,\mathrm{m}}}= & {} - A{\omega _{2,\mathrm{m}}} + B{i_{2,\mathrm{a}}} + C \end{aligned}$$

The Hamiltonian is

29 $$\begin{aligned} H = \left( {R_{\mathrm{a}}}{i_{2,\mathrm{a}}} + {K_{\mathrm{e}}}{\omega _{2,\mathrm{m}}}\right) {i_{2,\mathrm{a}}} + {\lambda _{2,1}}{\omega _{\mathrm{m}}} - {\lambda _{2,2}}\left( {A{\omega _{2,\mathrm{m}}} - B{i_{2,\mathrm{a}}}} \right) \end{aligned}$$

Similarly procedure is adopted to get the optimal control, and the costate equations are

30 $$\begin{aligned} {\dot{\lambda } _{2,1}}= & {} - \frac{ \partial H}{ \partial {\theta _{2,\mathrm{m}}}} = 0 \end{aligned}$$

31 $$\begin{aligned} {\dot{\lambda } _{2,2}}= & {} - \frac{{ \partial H}}{{ \partial {\omega _{2,\mathrm{m}}}}} \end{aligned}$$

The optimal control $i_{2,\mathrm{a}}^ *$ is derived also by setting the partial differential equation to zero; thus

32 $$\begin{aligned} i_{2,\mathrm{a}}^ * = \frac{{ - 1}}{{2{R_{\mathrm{a}}}}}\left( {{\lambda _{2,2}}B + {K_{\mathrm{e}}}{\omega _{2,\mathrm{m}}}} \right) \end{aligned}$$

while the subscript 2 denotes the corresponding variations of the location mode to distinguish the cruise start/stop mode. The boundary conditions of location mode are ${\omega _{2,\mathrm{m}}}(0) = {\omega _{2,\mathrm{m}}}({t_{\mathrm{f}}}) = 0, S({t_{\mathrm{f}}}) = {S_{\mathrm{f}}}, S({t_{\mathrm{f}}}) = {S_{\mathrm{f}}}$. The relation between the rotational angle of the motor ${\theta _{\mathrm{f}}}$ and the translational displacement of the robot ${S_{\mathrm{f}}}$ is

33 $$\begin{aligned} {\theta _{\mathrm{f}}} = \int _0^{{t_{\mathrm{f}}}} {{\omega _{2,\mathrm{m}}}\mathrm{d}t} = {S_{\mathrm{f}}}{i_{\mathrm{total}}}/\gamma \end{aligned}$$

Then, the optimal velocity is obtained by solving the above equations and the boundary conditions

34 $$\begin{aligned} {\omega _{\mathrm{m}}}\left( t \right)= {C_1}{e^{t/{\tau _{\mathrm{m}}}}} + {C_2}{e^{ - t/{\tau _{\mathrm{m}}}}} + {F_0} \end{aligned}$$

$$\begin{aligned}{C_1} &= \frac{{{e^{ - {t_{\mathrm{f}}}/{\tau _{\mathrm{m}}}}} - 1}}{{{e^{{t_{\mathrm{f}}}/{\tau _{\mathrm{m}}}}} - {e^{ - {t_{\mathrm{f}}}/{\tau _{\mathrm{m}}}}}}}{F_0}\nonumber \\ {C_2} &= \frac{{1 - {e^{{t_{\mathrm{f}}}/{\tau _{\mathrm{m}}}}}}}{{{e^{{t_{\mathrm{f}}}/{\tau _{\mathrm{m}}}}} - {e^{ - {t_{\mathrm{f}}}/{\tau _{\mathrm{m}}}}}}}{F_0}\nonumber \\ {F_0} &= \frac{{{S_{\mathrm{f}}}{i_{\mathrm{total}}}\left( {{e^{{t_{\mathrm{f}}}/{\tau _{\mathrm{m}}}}} - {e^{ - {t_{\mathrm{f}}}/{\tau _{\mathrm{m}}}}}} \right) }}{{\gamma \left( {{t_{\mathrm{f}}}\left( {{e^{{t_{\mathrm{f}}}/{\tau _{\mathrm{m}}}}} - {e^{ - {t_{\mathrm{f}}}/{\tau _{\mathrm{m}}}}}} \right) + 2{\tau _{\mathrm{m}}}\left( {2 - {e^{ - {t_{\mathrm{f}}}/{\tau _{\mathrm{m}}}}} - {e^{{t_{\mathrm{f}}}/{\tau _{\mathrm{m}}}}}} \right) } \right) }}\nonumber \\ {\tau _{\mathrm{m}}}= \, & {} {\sqrt{{A^2} + AB{K_{\mathrm{e}}}/{R_{\mathrm{a}}}} ^{ - 1}}\end{aligned}$$

here, ${C_1}$, ${C_2}$ and ${F_0}$ are decided by the initial boundary conditions, and ${\tau _{\mathrm{m}}}$ is the mechanical time constant. The optimal control $i_{2,\mathrm{a}}^ *$ can be also calculated from (25). Let $a = {t_{\mathrm{f}}}/{\tau _{\mathrm{m}}}$, and ${\tau _1} = t/{t_{\mathrm{f}}}$ be the reference time, and (34) can be reformed as

35 $$\begin{aligned} {\omega _{2,\mathrm{m}}}\left( t \right) = \frac{{{S_{\mathrm{f}}}{i_{\mathrm{total}}}}}{{\gamma {t_{\mathrm{f}}}}}a\frac{{\sinh \left( a \right) - \sinh \left( {a - {\tau _1}a} \right) - \sinh \left( {{\tau _1}a} \right) }}{{a\sinh \left( a \right) - 2\cosh \left( a \right) + 2}} \end{aligned}$$

Trzynadlowski had discussed the optimal velocity by plotting a figure [12]. We will find the extremum of the velocity analytically. When $a \rightarrow + 0$, by using the infinite series expansion ${e^a}$, (35) yields

36 $$\begin{aligned} \mathop {\lim }\limits _{a \rightarrow 0} {\omega _{2,\mathrm{m}}} = \frac{{6{\theta _{\mathrm{f}}}}}{{{t_{\mathrm{f}}}}}\left( {{\tau _1} - \tau _1^2} \right) \end{aligned}$$

and when $a \rightarrow + \infty$, (35) yields

37 $$\begin{aligned} \mathop {\lim }\limits _{a \rightarrow + \infty } {\omega _{2,\mathrm{m}}} = \frac{{S_{\mathrm{f}}}{i_{\mathrm{total}}}}{\gamma {t_{\mathrm{f}}}} \end{aligned}$$

**Fig. 7:**
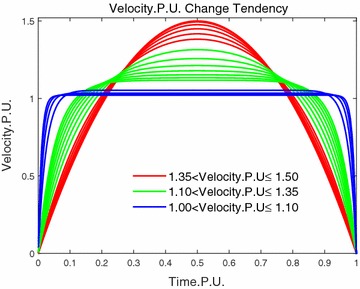
Optimal velocity changes under various of *a*

(36) and (37) can be explained as two extreme conditions for a certain system whose mechanical time constant ${\tau _{\mathrm{m}}}$ has been decided. Assume ${S_{\mathrm{f}}}$ keeps constant, and the final time ${t_{\mathrm{f}}}$ varies. When ${t_{\mathrm{f}}}$ is too short with respect to ${\tau _{\mathrm{m}}}$, the velocity profile will approach (36), but if ${t_{\mathrm{f}}}$ is too long with respect to ${\tau _{\mathrm{m}}}$, the velocity profile is getting close to (37). For better understanding, let ${\omega _{\mathrm{m}}}/({\theta _{\mathrm{f}}}/{t_{\mathrm{f}}})$ be the reference velocity and $t/{t_{\mathrm{f}}}$ be the reference time, and when $a = {t_{\mathrm{f}}}/{\tau _{\mathrm{m}}}$ varies, Fig. 7 is plotted as velocity per unit versus time per unit by using the parameters of Table 1.

#### Velocity constrains

It is known that each motor has its maximum speed limit; moreover, it also has a maximum continuous working speed ${\omega _{\mathrm{m,r}}}$ for practical use. Let ${k_{\mathrm{v}}}$ be the speed ratio and defined as

38 $$\begin{aligned} {k_{\mathrm{v}}} = \frac{{\omega _{\mathrm{m,r}}}\gamma {t_{\mathrm{f}}}}{ {{i_{\mathrm{total}}}{S_{\mathrm{f}}}}} \end{aligned}$$

From the above discussion, we will get $1 \le {k_{\mathrm{v}}} \le 1.5.$ Thus, when ${k_{\mathrm{v}}}$ is not belonging to this interval, then we need to formulate a new velocity profile. Because ${k_{\mathrm{v}}} = 1.5$, the velocity profile becomes parabola as indicated in (36), and we still adopt this parabolic curve as the velocity profile when ${k_{\mathrm{v}}} \ge 1.5$. Then, the velocity becomes

39 $$\begin{aligned} {\omega _{\mathrm{m}}}\left( t \right) = \frac{6{S_{\mathrm{f}}}{i_{\mathrm{total}}}}{{\gamma {t_{\mathrm{f}}}}}\left( {t/{t_{\mathrm{f}}} - {t^2}/t_{\mathrm{f}}^2} \right) \end{aligned}$$

As for the condition $0< {k_{\mathrm{v}}} < 1$, it means that the average speed ${S_{\mathrm{f}}}{i_{\mathrm{total}}}/\gamma {t_{\mathrm{f}}}$ that is given by operator has exceeded the maximum continuous working speed ${\omega _{\mathrm{m,r}}}$. Thus, the robot cannot move a displacement ${S_{\mathrm{f}}}$ within time ${t_{\mathrm{f}}}$. Generally, ${S_{\mathrm{f}}}$ is the destination and cannot change, and the other way is to adjust the time ${t_{\mathrm{f}}}$. From Fig. 7, we can see the region of this condition lies beneath the trapezoidal speed profile and we just follow this trend to form the velocity profile, which is

40 $$\begin{aligned} {\omega _{\mathrm{m}}}\left( t \right) = \left\{ \begin{array}{ll} {\omega _{\mathrm{m,r}}} - 4{\omega _{{\rm m,r}}}{\left( {t/{t_1} - 0.5} \right) ^2}&{}\qquad {0 \le t \le 0.5{t_1}} \\ {\omega _{\mathrm{m,r}}} &{}\qquad 0.5{t_1}< t \le {t_{\mathrm{f}}} - 0.5{t_1} \\ {\omega _{\mathrm{m,r}}} - 4{\omega _{\mathrm{m,r}}}{\left( {\left( {t - {t_{\mathrm{f}}}} \right) /{t_1} + 0.5} \right) ^2}&{}\qquad \left( {{t_{\mathrm{f}}} - 0.5{t_1}} \right) < t \le {t_{\mathrm{f}}} \end{array} \right. \end{aligned}$$

Between $[0,0.5{t_1}]$ the motor is accelerating the robot along the parabolic curve to the maximum continuous working speed of ${\omega _{\mathrm{m,r}}}$, then it moves at this speed for a while, and finally, it begins to decelerate the robot along the parabolic curve to complete the desired displacement. And ${t_1}$ and ${t_{\mathrm{f}}}$ can be derived by minimizing the total energy ${E_{\mathrm{in}}}$ with respect to the time *t*

41 $$\begin{aligned} {S_{\mathrm{f}}}= \,& {} \frac{\gamma }{i_{\mathrm{total}}}\int _0^{{t_{\mathrm{f}}}} {{\omega _{\mathrm{m,r}}}\mathrm{d}t} \end{aligned}$$

42 $$\begin{aligned} {E_{\mathrm{in}}}= & {} \int _0^{0.5{t_1}} {{V_{\mathrm{a}}}{i_{\mathrm{a}}}\mathrm{d}t} + \int _{{\tau _1}{t_1}}^{{t_{\mathrm{t}}} - 0.5{t_1}} {{V_{\mathrm{a}}}{i_{\mathrm{a}}}\mathrm{d}t} + \int _{{t_{\mathrm{f}}} - 0.5{t_1}}^{{t_{\mathrm{f}}}} {{V_{\mathrm{a}}}{i_{\mathrm{a}}}\mathrm{d}t} \end{aligned}$$

After discussing the above working conditions of the robot, we can formulate the optimal velocity selection strategy, which considers the velocity constrains, under location mode

43 $$\begin{aligned} {\omega _{\mathrm{m}}}\left( t \right) = \left\{ \begin{array}{ll} {\mathrm{Equation}\,(40)} &{}\quad {0 \le {k_{\mathrm{v}}}<1}\\ {\mathrm{Equation}\,(34)} &{}\quad {1 \le {k_{\mathrm{v}}} \le 1.5}\\ {\mathrm{Equation}\,(39)} &{}\quad {1.5 < {k_{\mathrm{v}}}}\\ \end{array} \right. \end{aligned}$$

Equation (43) guarantees the motor and the robot working in a continuous and safe region, and it is suitable for the pipe robot which will work in field environment. Judging maximum velocity problem does not exist in the cruise start/stop mode, because the control system will compare the input speed ${\omega _{\mathrm{f}}}$ with ${\omega _{\mathrm{m,r}}}$ directly and inform the operator to change the velocity, if ${\omega _{\mathrm{f}}}$ is larger than ${\omega _{\mathrm{m,r}}}$.

## Results and discussion

We have derived the energy-efficient control laws of the robot in cruise start/stop mode and location mode, respectively. In this section, simulations are performed to evaluate the proposed energy-efficient control law, and these results are compared with two benchmark methods. One is sinusoidal velocity function based on the computed torque control and is given by

44 $$\begin{aligned} S\left( t \right)= \, & {} \frac{{S_{\mathrm{f}}}}{2}\left( {1 - \cos \left( {\pi \frac{t}{t_{\mathrm{f}}}} \right) } \right) \end{aligned}$$

45 $$\begin{aligned} \omega \left( t \right)= \, & {} \frac{\pi {S_{\mathrm{f}}}}{{2\gamma {t_{\mathrm{f}}}}}\sin \left( {\pi \frac{t}{t_{\mathrm{f}}}} \right) \end{aligned}$$

The second benchmark method is the loss minimization control method that optimizes the armature loss $i_{\mathrm{a}}^2{R_{\mathrm{a}}}$ of the DC motor, which only considered $R_{\mathrm{a}}$.

**Table 1:** Parameters of the robot and motor [13]

Parameter	Value	Parameter	Value
*P*	20 W (rated)	*D*	0.19 m
${K_{\mathrm{t}}}$	0.0170 N m/A	$\alpha$	15°
${K_{\mathrm{e}}}$	0.0170 V s/rad	${T_{\mathrm{load}}}$	0.3 N m
${R_{\mathrm{a}}}$	1.17 Ω	${J_{\mathrm{eq}}}$	$4\times {10^{-5}}\,\mathrm{kg}\,\mathrm{m}^2$
${R_{\mathrm{h}}}$	212.6 Ω	$_{\mathrm{total}}$	111
$30{\omega _{\mathrm{m,r}}}/\pi$	6000 rpm	${c_{\mathrm{m}}}$	$2\times {10^{- 5}}$ N m s/rad

**Fig. 8:**
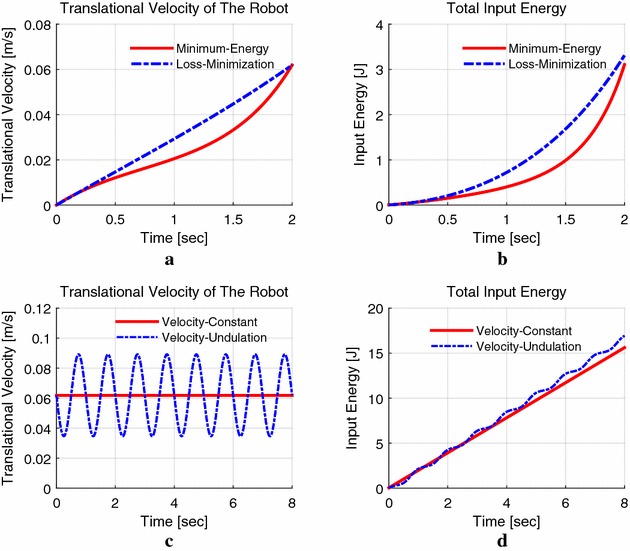
Cruise start mode with $\omega _{\mathrm{f}}=270$ rad/s, $t_{\mathrm{f}}=2$ s. **a** Velocity comparison, **b** energy dissipation, **c** velocity constant versus velocity undulation, **d** energy dissipation

Figure 8 shows the results of the two methods within the same time interval to reach a same speed that the operator inputs. The velocity and energy dissipation of minimum energy control are both lower than that of loss minimization control that only considers $R_{\mathrm{a}}$. Figure 8c shows the two conditions after the robot reached the speed of the cruise start mode: One is that the robot keeps the velocity constant, and the other is that the robot’s speed varies (we use a sinusoidal function in this figure). Figure 8d shows that the velocity variation needs more energy to keep the robot moving than that of keeping the velocity invariable. Thus, it is better to keep the speed of the robot stable in the pipe in order to save energy in the field environments.

**Fig. 9:**
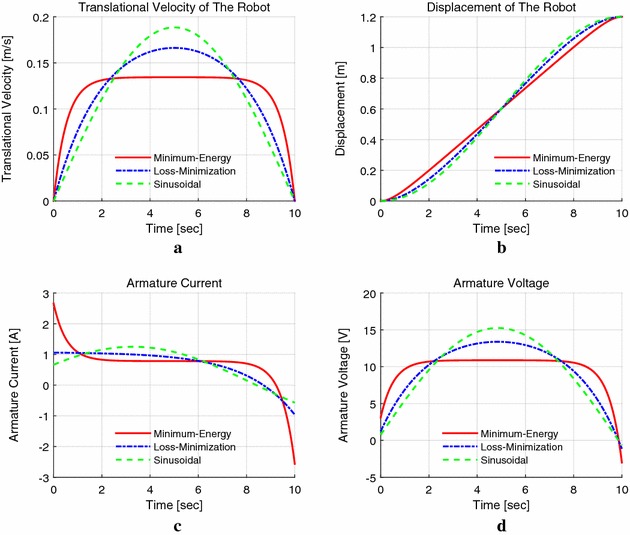
Location mode with $S_{\mathrm{f}}=1.2$ m, $t_{\mathrm{f}}=10$ s with $k_{\mathrm{v}}=1.20$. **a** Translational velocity, **b** displacement, **c** armature current, **d** armature voltage

In Fig. 9a the velocity of the minimum energy control is the lowest one, compared to that of the other two, whose speed exceeds the maximum continuous working speed 0.144 m/s for a short while. Figure 9a, c also shows that the speed of minimum energy control accelerates and decelerates rapidly and maintains a stable speed; this is convenient for the robot while checking the pipe, because a stable moving speed is reasonable for sensor to collect data; as for the other two methods, the speed varies during the whole operation time.

**Fig. 10:**
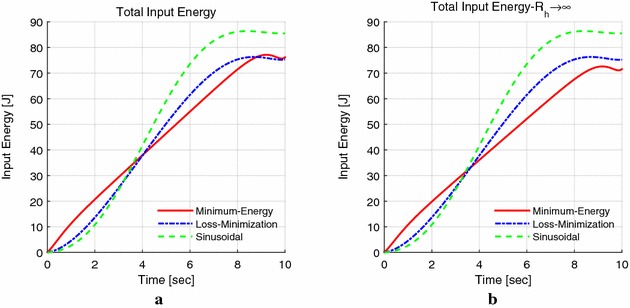
Results of the total energy consumption. **a** Results considering $R_{\mathrm{a}}$ and $R_{\mathrm{h}}$, **b** results of $R_{\mathrm{h}} \rightarrow \infty$ (only considering $R_{\mathrm{a}}$)

Figure 10a shows the energy dissipation of location mode in Fig. 9. The sinusoidal control causes the highest energy, while the loss minimization cost is the lowest. This is because in minimum energy control method, we have considered the armature resistance ${R_{\mathrm{a}}}$ and the equivalent resistance ${R_{\mathrm{h}}}$ of the power loss due to the air resistance of the rotor and power loss due to friction between the mechanical parts as shown in Fig. 3. Thus, the armature current is larger than that of only considering ${R_{\mathrm{a}}}$, when the robot works at the same combination of torque and velocity, which causes more electrical energy. Figure 10b is the condition that ${R_{\mathrm{h}}} \rightarrow \infty$, and the result shows the minimum energy control cost is the lowest energy. Therefore, when we consider ${R_{\mathrm{h}}}$, the total input energy will increase and may possibly greater than loss minimization control, but it gives a more accurate model and numerical results than that of only considering the armature resistance (see Table 2).

**Fig. 11:**
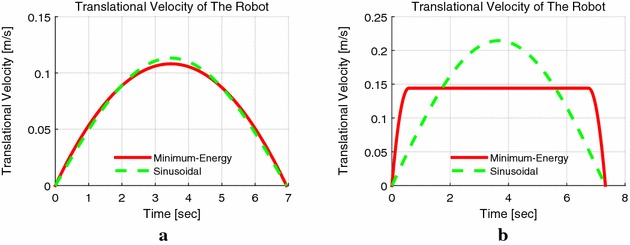
Velocity selection according to $k_{\mathrm{v}}$. **a** Velocity profiles under $S_{\mathrm{f}}=0.5$ m, $t_{\mathrm{f}}=6.94$ s, and $k_{\mathrm{v}}=2$, **b** velocity profiles under the required $S_{\mathrm{f}}=1$ m and $t_{\mathrm{f}}=1$ s that means $k_{\mathrm{v}}<1$, thus, the recalculated time $t_{\mathrm{f}}=7.33$ s and $t_1=1.16$ s

Figure 11 shows the velocity selection strategy according to the value of ${k_{\mathrm{v}}}$. When the value of ${k_{\mathrm{v}}}$ is greater than 1.5, the parabolic curve is selected as the velocity profile since it is the upper limit of the velocity per unit (see Fig. 7). While the value of ${k_{\mathrm{v}}}$ is lower than 1, which means even the maximum speed of motor does not satisfy the requirement of ${S_{\mathrm{f}}}$ and ${t_{\mathrm{f}}}$, the velocity is generated according to (40), which minimizes the total energy as well. From the above, we can see that the minimum energy control causes lower energy dissipation and provides more accurate numerical results, compared to that of the loss minimization control only considering armature resistance. The summary of energy dissipations is listed in Table 2.

**Table 2:** Summary of total input energy

*S*_f_ (m)	*t*_f_ (s)	I: $\int _0^{{t_{\mathrm{f}}}} {{V_{\mathrm{a}}}{i_{\mathrm{a}}}} \mathrm{d}t$	II: $\int _0^{{t_{\mathrm{f}}}} {{i_{\mathrm{a}}}^2{R_{\mathrm{a}}}} \mathrm{d}t$	III: $\int _0^{{t_{\mathrm{f}}}} {{V_{\mathrm{a}}}{i_{\mathrm{a}}}} \mathrm{d}t$
0.3 m	4	14.79 (14.02)	14.37	15.65
1.2 m	10	76.09 (71.46)	75.28	85.53
2.0 m	15	134.91 (126.50)	132.83	155.18
Figure 8b		3.10	3.31	14.12
Figure 11a		23.30 (19.70)	21.65	23.91
Figure 11b		83.82	N/A	86.40

*I* refers to the minimum energy control that considers $R_{\mathrm{a}}$ and $R_{\mathrm{h}}$, while the numbers in the bracket denote the results that $R_{\mathrm{h}} \rightarrow \infty$; *II* refers to the loss minimization control that only considers $R_{\mathrm{a}}$; *III* refers to the computed torque control that considers $R_{\mathrm{a}}$ and $R_{\mathrm{h}}$

## Conclusion

This paper considers a more accurate motor model and uses total input energy as the cost function to generate energy-efficient control laws for a pipe inspection robot. This pipe robot has two working modes: driving mode and detecting mode. Robot needs to keep a speed to move or move a distance to check the pipe; thus, we propose two types control: One is cruise start/stop control, and the other is location control. For the cruise mode and location mode, we have derived the optimal velocity and propose a velocity selection strategy according to the $k_{\mathrm{v}}$. This velocity selection strategy can guarantee the motor work in safe region, which also means decreasing the total input energy consumption, and can treat all the combinations of the $S_{\mathrm{f}}$ and $t_{\mathrm{f}}$. Results show that this method indeed saves the energy dissipation with the commonly used method, and provide more accurate model compared with the loss minimization control.
